# Genome-wide analysis of miRNAs in the ovaries of Jining Grey and Laiwu Black goats to explore the regulation of fecundity

**DOI:** 10.1038/srep37983

**Published:** 2016-11-29

**Authors:** Xiangyang Miao, Qingmiao Luo, Huijing Zhao, Xiaoyu Qin

**Affiliations:** 1 Institute of Animal Sciences, Chinese Academy of Agricultural Sciences, Beijing 100193, China

## Abstract

Goat fecundity is important for agriculture and varies depending on the genetic background of the goat. Two excellent domestic breeds in China, the Jining Grey and Laiwu Black goats, have different fecundity and prolificacies. To explore the potential miRNAs that regulate the expression of the genes involved in these prolific differences and to potentially discover new miRNAs, we performed a genome-wide analysis of the miRNAs in the ovaries from these two goats using RNA-Seq technology. Thirty miRNAs were differentially expressed between the Jining Grey and Laiwu Black goats. Gene Ontology and KEGG pathway analyses revealed that the target genes of the differentially expressed miRNAs were significantly enriched in several biological processes and pathways. A protein-protein interaction analysis indicated that the miRNAs and their target genes were related to the reproduction complex regulation network. The differential miRNA expression profiles found in the ovaries between the two distinctive breeds of goats studied here provide a unique resource for addressing fecundity differences in goats.

The domestic goat (*Capra hircus*) is raised all over the world and is a great sector in the consumer market, providing humans with meat, skin, fur, fiber, and so on. The Laiwu Black and Jining Grey goats are two excellent local breeds in Shandong, China. The Jining Grey goat is an excellent local breed in China that possesses the characteristics of high prolificacy and year-round estrus. The fecundity of the Laiwu Black goat is much lower than that of the Jining Grey goat^1^. Specifically, the mean litter size for the Jining Grey goats is 2.94^2^. In contrast, the fecundity of the Laiwu Black goat is only 1.64^3^. In the goat industry, these reproductive traits are largely important because even a slight increase in litter size can lead to a large profit^4^.

Previous studies indicate that the bone morphogenetic protein receptor-1B (BMPR1B) gene regulates the fecundity and ovulation rate of sheep^5^,^6^,^7^. The goat genome sequence was recently resolved^8^,^9^, and despite the progress made in goat fecundity studies, the genes involved in the regulation of fecundity in goats are largely unknown. Thus, it may not be possible to improve fecundity at this point, and this is mostly due to the fact that reproductive traits are complex quantitative traits involving multiple genes, loci, and interactions. To beter understand goat fecundity, it is necessary to analyze the combined effect of multiple genes or loci on reproductive traits. Therefore, it is neceasary to identify more functional genes, to clarify the molecular mechanisms of actions and to identify regulatory networks that might be involved in goat fecundity^4^.

miRNAs are endogenous small non-coding RNAs that play an important role in gene regulation in animals and plants by pairing to the mRNAs of protein-coding genes to direct their posttranscriptional repression^10^,^11^. Generally, miRNAs are initially transcribed by RNA polymerase II and mature miRNAs form the RNA-induced silencing complex (RISC) with other components, such as dicer. Gene silencing occurs through the paring of complementary mRNA and miRNA, which, thus, leads to mRNA degradation or the prevention of translation^11^. More and more evidence suggests that hundreds of miRNAs affect a large fraction of the transcriptome and a broad range of biological processes and signaling pathways, such as the regulation of the transforming growth factor-β, Wnt, Notch, and epidermal growth factor signaling pathways^12^,^13^.

Recently, the RNA-Seq approach, which is a powerful tool based on next-generation deep-sequencing technologies, was utilized for RNA analysis^14^. The sequence reads from these analyses are individually mapped to the source genome and counted to obtain the number and density of reads corresponding to the RNA from each known exon, splice event or new candidate gene^15^. This technique has been successfully applied in multiple organisms for the genome-wide analysis of RNAs, such as yeast^16^, sheep^17^,^18^,^19^,^20^,^21^, and human^22^. The current study aimed to characterize distinct gene regulation between two domestic goats raised in China using genome-wide miRNA profiling via high-throughput deep RNA sequencing (RNA-Seq) technology.

## Results

### Mapping the small RNA reads

The small RNA reads obtained from the ovaries of the two goat breeds were first mapped using the goat miRBase database, and the resulting mapping rate was 92.1% (1.86 of 2.02 M) and 93.7% (2.47of 2.61 M) of total clean reads for the Jining Grey goats and the Laiwu Black goats, respectively (Table 1). Additional mapping to other species was attempted on the remaining reads, and a ~0.4% mapping rate was obtained that covered 297 and 278 miRNAs from other species for the Jining Grey and Laiwu Black goats, respectively (Table 2). For reads that did not map in the miRBase databases, the miRNA prediction process was performed to identify pre-mature miRNAs and to discover potential novel miRNAs.

### Novel miRNA prediction

The novel miRNAs were predicted using Mireap software^23^, and the predicted miRNAs were all essentially with a hair-pin structure. The predicted miRNAs were first mapped to the goat database and were subsequently mapped to the sheep, cow, pig, rat, mouse, and human miRBase databases for annotations. As shown in Table 1, 56.1% (10,383/18,499) and 56.3% (11,355/20,186) of the predicted miRNA reads in the Jining Grey and Laiwu Black goat samples, respectively, were mapped to the miRBase databases corresponding to these species.

### Length distribution and base preference of the miRNAs

The length of the mapped miRNAs in this study was around 22 nt. Previous studies suggest that the miRNAs with a base U at the 5′ terminus are more conserved than miRNAs with a non-U base at the 5′ terminus^24^. We analyzed the base at the 5′ terminus of the miRNAs identified, and the results showed that U is the most common base in both of the goat breeds.

### Differential regulation of the miRNAs between the two breeds of goats

A total of 5254 miRNAs were identified in the two goat samples, of which 603 (314 mature and 289 predicted, Table S1) had at least 10 read counts in the two goat samples. The expression level of these 603 conserved and novel miRNAs was assessed by a differential analysis, a downstream target prediction and biological annotation enrichment analyses. At a false discovery rate (FDR) <0.05 and an absolute value of log_2_ (fold change) > = 1, 30 miRNAs (22 mature and 8 predicted) were defined as differentially expressed miRNAs between the Laiwu Black and Jining Grey goats (Table 3), of which 10 miRNAs were down-regulated and 20 were up-regulated in the Jining Grey goat as compared with the Laiwu Black goat.

### Prediction of the miRNA targets

The programs TargetFinder^25^ and miRanda^26^ were used to assess the 3′-UTR regions of the potential target genes. A total of 15,848 gene targets without redundancy were prediced from the 30 differential miRNAs (30,172 pairs) with a free enengy (ΔG) of less than or equal to −15.0, with 1–7 miRNAs targeting a single gene or 17–4357 genes targeted by a single miRNA. Of these 15, 848 gene targets, known miRNAs gene targets without redundancy were 13,699, and predicted miRNAs gene targets without redundancy were 6,703 (Table S2).

### Gene Ontology and KEGG pathway analysis

**Figure 1:**
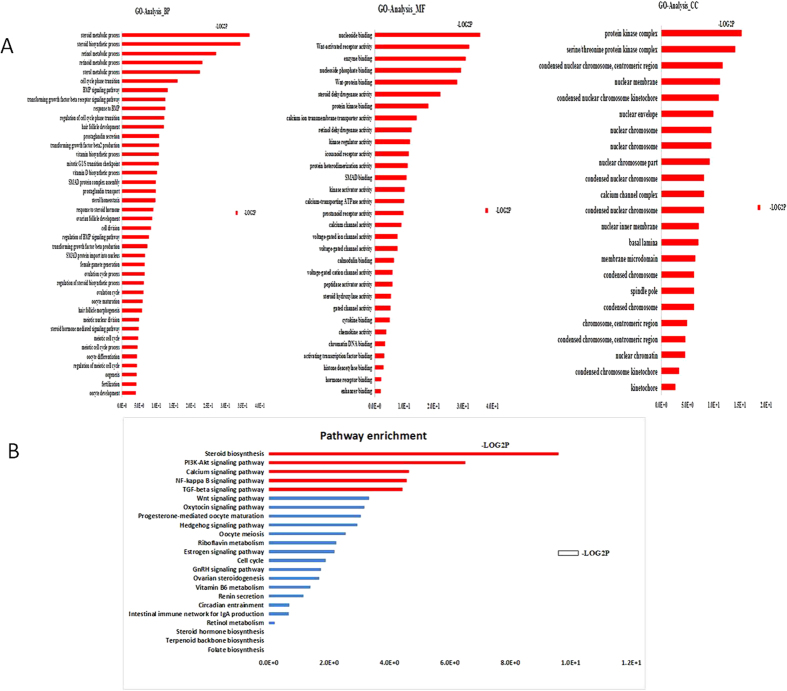
Summary of the gene ontology and KEGG pathway analyses of the targets of the differentially expressed miRNAs. (**A**) Gene ontology and (**B**) KEGG pathway. The processes and pathways in red are significant.

**Figure 2:**
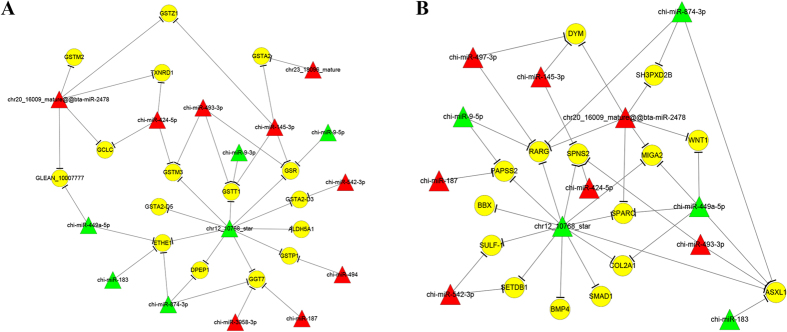
Two specific miRNA-mRNA regulation networks. (**A**) glutathione metabolic process and (**B**) bone development. The yellow circle indicates mRNA. Red and green triangles represent miRNAs. Red and green represents up and down regulation respectively.

The predicted miRNA-targeted genes were further analyzed by Gene Ontology (GO) and the Kyoto Encyclopedia of Genes and Genomes (KEGG) pathway analysis. The target genes that interact with conserved and predicted miRNAs were analyzed separately, with regard to the enrichment of the GO terms and the KEGG pathway analysis. For the mature miRNA-targeted genes, there were no significantly enriched GO terms and KEGG pathways. In contrast, the predicted miRNA-targeted genes were enriched significantly in 28 biological processes and 2 KEGG pathways (Fig. 1). Among the 28 biological processes, 19 were associated with reproduction, such as steroid biosynthetic process, the BMP signaling pathway, and the transforming growth factor beta receptor signaling pathway (Figs 1A and 2A). Glycosaminoglycans that are localized to the extracellular matrix of bone are thought to play a key role in mediating aspects of bone development. The two enriched KEGG pathways were the TGF-β signaling pathway and steroid biosynthesis (Figs 1B and 2B). Indeed, these functional annotation results demonstrate that this database contains clues that might lead to uncovering potential regulators of fecundity between these 2 breeds of goats.

### Interaction networks between miRNAs and the target genes related to reproduction

**Figure 3:**
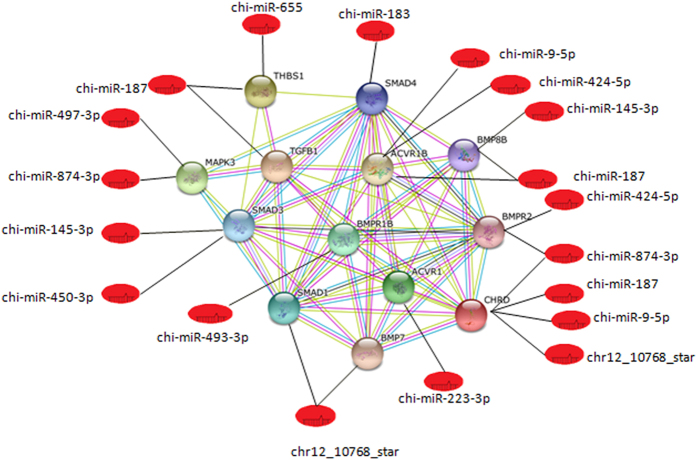
Network of the miRNAs with the targeted genes related to reproduction.

Given the fact that we are interested in exploring the fecundity differences between these 2 breed of goats, the relationships between the differentially expressed miRNAs and the targeted genes related with reproduction are described based on the STRING database analysis. Figure 3 demonstrates that the relationships between the miRNAs and the genes BMPR 1B, SMAD1, BMP7, ACVR1, CHRD, BMPR2, BMP88, ACVR18, SMAD4, THBS1, TGFB1, MAPK3 and SMAD3, which are related to reproduction, are complicated. The BMPR1B gene, which is regulated by chi-miR-493-3P alone, is a very important finding. In addition, chi-miR-187 regulates four genes, including TGFB1, THBS1, ACVR18 and BMP88, and chr12_10768_star regulates three genes, including CHRD, SMAD1 and BMP7. In addition, chi-miR-874-3P regulates three genes, including MAPK3, BMPR2 and CHRD. Therefore, chi-miR-187, chr12_10768_star and chi-miR-874-3P might play an important role in reproductive regulation processes.

### qRT-PCR validation of RNA-Seq data

**Figure 4:**
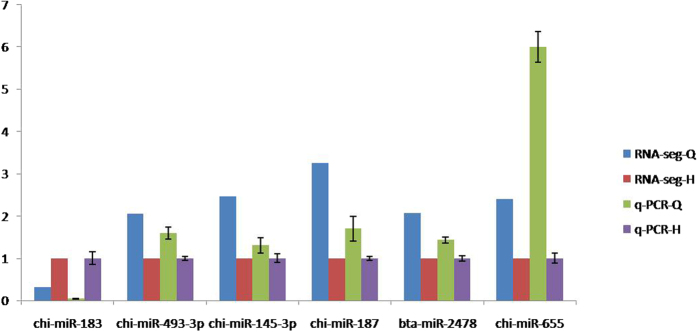
qPCR validation of 6 miRNAs. The gene U6 was used as the reference gene.

To confirm the RNA-Seq data, 6 differentially expressed miRNAs were examined by qRT-PCR, and these data were consistent with the above RNA-Seq results (Table 4 and Fig. 4). The genes potentially targeted by the 6 miRNAs are involved in diverse cellular activities, such as signal transduction, kinase, motor activity, transport, metabolic process and DNA binding.

## Discussion

With the use of RNA-Seq technology, here, we performed a genome-wide analysis on the miRNAs in Jining Grey and Laiwu Black goats and characterized 30 differentially expressed miRNAs. Because these two breeds of goats demonstrate different fecundity, the differentially regulated miRNAs may contribute to this process. Previous studies indicate that several miRNAs are important for the follicular-luteal transition in the ruminant ovary and for fetal gonadal development^27^,^28^. Homologs of the identified differentially expressed miRNAs function in various cellular activities. For instance, miR-9-3p is an important regulatory factor in the osteoblastic differentiation of mouse iPS cells and also targets β1 integrin to sensitize claudin-low breast cancer cells to MEK inhibition^29^,^30^. miR-449a regulates cell proliferation and causes Rb-dependent cell cycle arrest and senescence^31^,^32^. Mature miR-183 promotes 3T3-L1 adipogenesis through the inhibition of the canonical Wnt/β-catenin signaling pathway by targeting LRP6^33^. miR-542-3p also has many important functions, including the induction of growth arrest through the survival pathway, the suppression of osteoblast cell proliferation and differentiation, the targeting of BMP-7 signaling and the inhibition of bone formation^34^,^35^. Given the broad roles of the miRNA homologs, the differentially expressed miRNA molecules might also potentially be involved in the regulation of goat growth and development.

miRNAs function mainly by pairing with mRNAs, and by an *in silico* analysis, we predicted the potential target genes for the differentially expressed miRNAs identified in our RNA-Seq analysis. Using the predicted target genes, the Gene Ontology (GO) enrichment analysis revealed several significantly enriched biological processes that were associated with the steroid biosynthetic process, the BMP signaling pathway and the transforming growth factor beta receptor signaling pathway (Fig. 1). The glutathione metabolic process is involved in a variety of protective processes, and we found that, in this process, 15 differentially expressed miRNAs potentially target 16 genes (Fig. 2A), which include the homologs of miR-9-3p, miR-183, miR-449a and miR-542-3p, which were mentioned in the prior section. In addtion, more favorable cellular processes in the ovaries, such as the steroid biosynthetic process, the BMP signaling pathway, the transforming growth factor beta receptor signaling pathway, contribute to the high prolificacy observed in the Jining Grey goat. Moreover, among the differentially expressed miRNAs that were identified to be involved in the glutathione metabolic process, 15 of the predicted target genes are also involved in the bone development (Fig. 2B). A recent study reported^36^ that certain genes in ovaries were involved in cartilage development and bone healing, which is consistent with our findings. The KEGG pathway analysis was consistent with the GO analysis, indicating that the miRNA targeted genes were significantly enriched for glutathione metabolism and the steroid biosynthesis pathways.

Moreover, the relationship between the miRNAs and the genes BMPR1B, SMAD1, BMP7, ACVR1, CHRD, BMPR2, BMP88, ACVR18, SMAD4, THBS1, TGFB1, MAPK3 and SMAD3, which are related with reproduction^37^, is complicated. The BMPR1B gene, which is regulated by chi-miR-493-3P alone, was a very important finding. BMPR1B (FecB), was the first major prolificacy gene identified in sheep. The FecB locus is autosomal with a codominant expression and has an additive effect for ovulation rate when it is associated with a mutation (Q249R) in the BMPR1B gene^38^,^39^. BMPR1B is a member of the bone morphogenetic protein (BMP) receptor family of transmembrane serine/threonine kinases. BMPs are involved in endochondral bone formation and embryogenesis. BMPR1B is a member of the family of receptors for transforming growth factor (TGF) ligands, such as TGF, activins, BMPs and growth and differentiation factors (GDFs), which play a role in folliculogenesis^40^.

In conclusion, we identified 30 differentially expressed miRNAs in the ovaries of the two goats and most of these had defined homologs that were key miRNAs related to reproduction. This genome-wide miRNA analysis study will serve as a resource for understanding goat proliferacy. The differential miRNAs identified here are predicted to contribute to different prolificacies of the two goat breeds through a number of biological processes and pathways. In particular, Chi-miR-187, chr12_10768_star, and chi-miR-874-3P may play an important role in the reproductive regulation processes. This study provides a unique resource to address important issues related to goat fecundity, which will aid in elucidating the specific mechanisms responsible for differential gene expression in the ovaries between two distinctive breeds of goats. Moreover, these data also highlight the important roles of miRNAs during genetic regulation in different goat breeds.

## Materials and Methods

### Ethics statement

All of the experiments were performed in accordance with the relevant guidelines and regulations and were approved by the Institutional Animal Care and Use Committee of Institute of Animal Sciences, Chinese Academy of Agricultural Sciences.

### Goat sample preparation

All of the samples in this study were from female adult goats between the ages of 1.5 to 2 years old. The age-matched healthy female Jining Grey goats and female Laiwu Black goats (5 per breed) were obtained from the Qingdao Aote Farm (Shandong, China). The goats were treated with intravaginal sponges (Intervet, Mexico) to achieve estrous synchronization as previously described^41^. Pregnant mare serum gonadotropin (PMSG) (Ningbo Sansheng Pharmaceutical Co., LTD., Zhejiang, China) was injected after the removal of the sponge. Estrus was checked by observing the goats’ reactions to an adult male goat every 12 h after the sponge was removed. The female goats were determined to be in estrus when they showed standing estrous behavior. The goats were sacrificed 4–5 hours after estrus was determined, and the whole ovaries were excised. The samples were collected to obtain ovulation points on the surface of the ovaries. All of the samples were immediately snap-frozen in liquid nitrogen and stored at −70 °C for RNA extraction^41^.

### Construction of small RNA libraries and sequencing

Total RNA was extracted from ovaries with better ovulation points on the surfaces from 5 Jining Grey and 5 Laiwu Black goats using Trizol (Life Technologies/Invitrogen, California, USA) according to the manufacturer’s instructions^42^. The extracted RNA from each breed was pooled. The quality and quantity of the RNA samples were assessed on a Bioanalyzer 2100 system using an RNA 6000 Nano kit (Agilent Technologies, Palo Alto, CA). After the total RNA was extracted, two small RNA libraries were prepared as described in the Illumina^®^ TruSeq™ Small RNA Sample Preparation protocol. First, the 3′ adaptor and 5′ adaptor were ligated to the RNA by T4 RNA ligase, and then, reverse transcription was carried out with SuperScriptII reverse transcriptase (Invitrogen) to generate the cDNA. The resulting cDNA was then amplified to generate the small RNA library. The DNA size and the purity of the cDNA library were checked using a high sensitivity DNA 1000 kit on a Bioanalyzer 2100 system (Agilent Technologies, Santa Clara, CA), and the quantification of the cDNA libraries was performed with Qubit™ dsDNA HS kit on a Qubit^®^ 2.0 Fluorometer (Life Technologies, CA, USA). The cDNA libraries were then subjected to single-end sequencing on the Illumina Genome AnalyzerIIx (GAIIx) using the proprietary Solexa sequencing-by-synthesis method at the Shanghai Biotechnology Corporation (Shanghai, China) according to the manufacturer’s recommended cycling parameters. The image analysis and the base calling were performed with the Illumina built-in SCS2.8/RTA1.8 software.

### Workflow of the bioinformatics analysis

**Figure 5:**
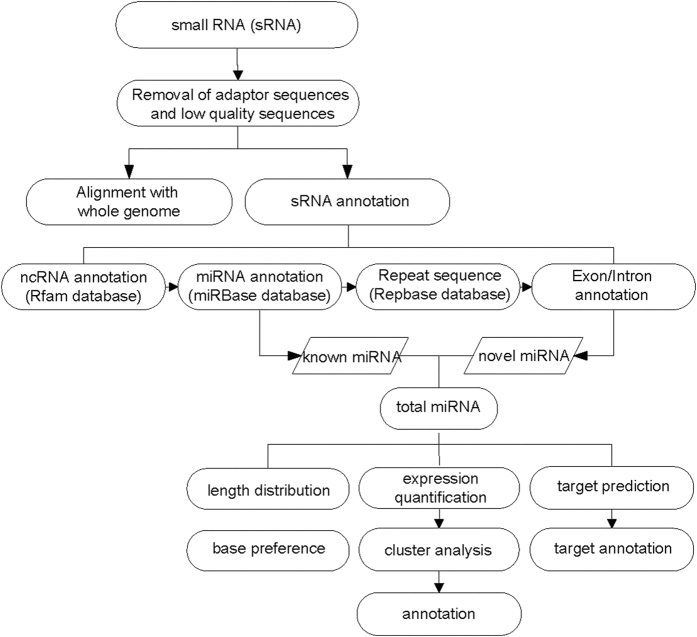
Workflow of the bioinformatics analysis of the small RNA sequencing results. Quality control was performed followed by further analysis.

The workflow of bioinformatics analysis of the RNA-Seq results is shown in Fig. 5. Briefly, the adaptor sequences and the low quality sequences were removed from the original reads by fastx (fastx_toolkit-0.0.13.2) (http://hannonlab.cshl.edu/fastx_toolkit/), and the quality read data were aligned with the whole reference genome and were compared with multiple databases for the annotation of the ncRNA, miRNA, repeat sequences, and exon/intron sequences. The differential expression of the miRNAs was identified using IDEG6 software^43^. For the unannotated small RNA sequences, a miRNA prediction process was carried out, and then, the novel miRNAs and the targets of the differentially expressed miRNAs were annotated.

### Programs used for the bioinformatics analysis

To predict potential novel miRNAs, Mireap software^23^ was used to analyze the unannotated reads. The inverted repeats (step loops or hairpin structure) described by Jones-Rhoades and Bartel^44^ were searched, and the secondary structure of the inverted repeat was predicted by RNA fold^45^. To predict the targets of the miRNAs, the programs TargetFinder^25^ and miRanda^26^ were used. The differentially expressed miRNAs were identified using IDEG6 software^43^. A rigorous significance test for the digital gene expression profiling was used^46^. The differentially expressed gene contigs were analyzed by gene ontology (GO)^47^. Three structured controlled vocabularies (ontologies), including cellular component, molecular function, and biological process, were used to create a consistent description of the gene products as previously described^48^,^49^. Like the GO enrichment, the association of the genes with different pathways was computed using the Kyoto Encyclopedia of Genes and Genomes (KEGG) database (http://www.genome.jp/kegg)^50^,^51^. Moreover, based on the String database (http://string-db.org/), the interaction networks of the miRNAs and their target genes, related with reproduction, were predicted to draw relationships between the miRNAs and their target genes.

### Validation of RNA-Seq data

To validate the RNA-Seq results, the expression of several of the miRNAs was confirmed by quantitative real-time RT-PCR. Briefly, the cDNA for the miRNA was produced using a miScript II Reverse Transcription Kit (Qiagen, US) in a GeneAmp^®^ PCR System 9700 (Applied Biosystems, USA). Next, the cDNA was used as the template for the qPCR, which was performed using a SYBR Green I Master (Roche, Swiss). The primer sequences were designed in the laboratory and were synthesized by Generay Biotech (Generay, PRC) and are based on the mRNA sequences obtained from the NCBI database. The gene U6 was used as the internal control, and the expression levels were calculated using the 2 delta delta Ct method (2−ΔΔCt).

## Additional Information

**How to cite this article**: Miao, X. *et al*. Genome-wide analysis of miRNAs in the ovaries of Jining Grey and Laiwu Black goats to explore the regulation of fecundity. *Sci. Rep.* **6**, 37983; doi: 10.1038/srep37983 (2016).

**Publisher's note:** Springer Nature remains neutral with regard to jurisdictional claims in published maps and institutional affiliations.

## Supplementary Material

Supplementary Information

Supplementary Table S1

Supplementary Table S2

## Figures and Tables

**Table 1:** Reads mapping statistics.

Category	Jining Grey	%	Laiwu Black	%
Mapped in goat miRBase	1,861,671	92.05	2,446,554	93.70
Mapped in other species	7,305	0.36	7,736	0.30
Novel annotated	10,383	0.51	11,355	0.43
Novel non-annotated	8,116	0.40	8,831	0.34
Total mapped	1,887,475	93.33	2,474,476	94.77
Unmapped	134,885	6.67	136,694	5.23
Total celan reads	2,022,360	100.00	2,611,170	100.00

**Table 2:** Species of mapped miRNAs.

	Category	Jining Grey	Laiwu Black
mature	goat	383	373
	sheep	9	7
	cow	81	76
	pig	22	17
	rat	33	29
	mouse	75	69
	human	77	80
predict	non-annotated	1146	1316
	sheep	15	17
	cow	112	121
	pig	33	32
	rat	8	7
	mouse	7	10
	human	14	10
	Total mapped	2015	2164

**Table 3:** 30 differentially expressed miRNAs.

miRNA Name	Normalized counts - Jining Grey	Normalized counts - Laiwu Black	Log2FC	FDR	Style	Raw counts - Jining Grey	Raw counts - Laiwu Black
chr14_11964_mature	0	19.50487369	−20	6.12E-05	down	0	20
chi-miR-9-3p	19.48231021	152.1380148	−2.965144	1.22E-11	down	19	156
chi-miR-9-5p	51.26923741	285.7463996	−2.47857	1.29E-11	down	50	293
chr12_10768_star	14.35538647	78.01949477	−2.442242	3.98E-06	down	14	80
chr18_14930_mature	7.177693237	30.23255422	−2.074511	0.019925	down	7	31
chi-miR-183	31.78692719	102.4005869	−1.687719	0.000535	down	31	105
chi-miR-449a-5p	212.2546429	578.319505	−1.446071	4.46E-05	down	207	593
chr4_3193_mature	25.6346187	69.24230161	−1.43356	0.021011	down	25	71
chr4_3446_mature	44.09154417	112.1530237	−1.346895	0.008989	down	43	115
chi-miR-874-3p	48.19308316	112.1530237	−1.218571	0.027479	down	47	115
chi-miR-30f-5p	252.244648	124.8311916	1.014845	0.04378	up	246	128
chi-miR-3958-3p	253.2700328	124.8311916	1.020698	0.041048	up	247	128
chi-miR-493-3p	235.8384921	115.0787548	1.035178	0.038424	up	230	118
chr20_16009_mature@@bta-miR-2478	280.955421	135.5588722	1.051422	0.026344	up	274	139
chi-miR-450-5p	3051.545011	1434.58346	1.088908	0.007197	up	2976	1471
chi-miR-202-5p	147.6554037	67.29181424	1.133731	0.030467	up	144	69
chi-miR-424-5p	3025.910392	1378.99457	1.133752	0.00353	up	2951	1414
chi-miR-136-3p	1258.147086	566.6165808	1.150856	0.002616	up	1227	581
chi-miR-494	568.0631505	251.6128706	1.174846	0.002685	up	554	258
chi-miR-655	91.25924259	38.0345037	1.262662	0.030556	up	89	39
chr23_18096_mature	173.2900224	72.16803267	1.263757	0.005829	up	169	74
chi-miR-369-3p	97.41155107	39.98499107	1.284634	0.021533	up	95	41
chi-miR-145-3p	1201.750925	487.6218423	1.301303	0.000224	up	1172	500
chi-miR-542-3p	1539.102507	620.2549835	1.311156	0.000173	up	1501	636
bta-miR-132	156.8838665	61.44035213	1.352439	0.002894	up	153	63
chi-miR-450-3p	141.5030952	52.66315897	1.425968	0.001808	up	138	54
chi-miR-223-3p	95.36078158	34.13352896	1.482206	0.004285	up	93	35
chi-miR-187	37.93923568	11.70292422	1.696822	0.037147	up	37	12
chi-miR-497-3p	60.49770014	10.72768053	2.495542	2E-05	up	59	11
chr11_10274_mature	16.40615597	1.950487369	3.072331	0.022057	up	16	2

**Table 4:** Validation of RNA-Seq data by RT-PCR.

	RNA-Seq-Grey goat	RNA-Seq-Black goat	q-PCR-Grey goat	q-PCR-Black goat	q-PCR-Grey goat SD	q-PCR-Black goat SD
chi-miR-183	0.310417431	1	0.0416	1.0000	0.004	0.1503
chi-miR-493-3p	2.049366037	1	1.593	1.0000	0.1396	0.04933
chi-miR-145-3p	2.464514141	1	1.307	1.0000	0.1798	0.1026
chi-miR-187	3.241859467	1	1.7	1.0000	0.294	0.05
bta-miR-2478	2.072571248	1	1.43	0.9967	0.0781	0.05783
chi-miR-655	2.399380397	1	5.997	1.0000	0.363	0.115
