# Supplementary material for: Genome-wide analysis of miRNAs in the ovaries of Jining Grey and Laiwu Black goats to explore the regulation of fecundity
Source: Sci Rep. 2016 Nov 29;6:37983. doi: 10.1038/srep37983 (PMC5126701; doi:10.1038/srep37983)
Supplement: Supplementary Information [file srep37983-s1.doc]

Supplementary Information

**Genome-wide analysis of miRNAs in the ovaries of Jining Grey and Laiwu Black goats to explore the regulation of fecundity**

Xiangyang Miao^*^ Qingmiao Luo Huijing Zhao Xiaoyu Qin

Institute of Animal Sciences, Chinese Academy of Agricultural Sciences, Beijing, 100193, China

^*^Corresponding author: Xiangyang Miao Institute of Animal Sciences, Chinese Academy of Agricultural Sciences, Beijing, 100193, China, Tel: 86-10-62895663, Fax: 86-10-62895663 (China), E-mail: miaoxy32@163.com, [mxy32@sohu.com](mailto:mxy32@sohu.com)

**Table S1** 603 miRNAs with 10 or more counts in the two samples

**Table S2** 6703 predicted miRNA gene targets without redundancy
